# Indoleamine 2,3 dioxygenase (IDO) level as a marker for significant coronary artery disease

**DOI:** 10.1186/s12872-021-02140-0

**Published:** 2021-07-26

**Authors:** Nattawut Wongpraparut, Ploy Pengchata, Sudarat Piyophirapong, Pariya Panchavinnin, Rungtiwa Pongakasira, Noppadol Arechep, Kanda Kasetsinsombat, Kittipong Maneechotesuwan

**Affiliations:** 1 Division of Cardiology, Department of Medicine, Faculty of Medicine Siriraj Hospital, Mahidol University, 2 Wanglang Road, Bangkoknoi, Bangkok, 10700 Thailand; 2 Department of Clinical Pathology, Faculty of Medicine Siriraj Hospital, Mahidol University, Bangkok, Thailand; 3 Her Majesty’s Cardiac Center, Faculty of Medicine Siriraj Hospital, Mahidol University, Bangkok, Thailand; 4 Department of Pharmacology, Faculty of Medicine Siriraj Hospital, Mahidol University, Bangkok, Thailand; 5 Division of Respiratory Diseases and Tuberculosis, Department of Medicine, Faculty of Medicine Siriraj Hospital, Mahidol University, Bangkok, Thailand

**Keywords:** Indoleamine 2,3 dioxygenase level, IDO, Marker, Significant coronary artery disease

## Abstract

**Background:**

Indoleamine 2,3 dioxygenase (IDO), the rate-limiting enzyme in the kynurenine (Kyn) pathway of tryptophan (Trp) degradation, is modulated by inflammation, and is regarded as a key molecule driving immunotolerance and immunosuppressive mechanisms. Little is known about IDO activity in patients with active coronary artery disease (CAD).

**Methods:**

We prospectively enrolled patients who were scheduled to undergo coronary angiography. Measurement of IDO, high-sensitivity troponin T (hs-TnT), and high-sensitivity C-reactive protein (hs-CRP) levels was performed at baseline, and IDO activity was monitored at the 6-month follow-up.

**Results:**

Three hundred and five patients were enrolled. Ninety-eight patients (32.1%) presented with recent acute coronary syndrome (ACS). Significant difference in IDO, kynurenine, and hs-TnT between patients with and without significant CAD was observed. Baseline IDO activity, kynurenine level, and hs-TnT level were all significantly higher in significant CAD patients with 3-vessel, 2-vessel, and 1-vessel involvement than in those with insignificant CAD [(0.17, 0.13, and 0.16 vs. 0.03, respectively; *p* = 0.003), (5.89, 4.58, and 5.24 vs. 2.74 µM/g, respectively; *p* = 0.011), and (18.27, 12.22, and 12.86 vs. 10.89 mg/dL, respectively; *p* < 0.001)]. One-year mortality was 3.9%. When we compared between patients who survived and patients who died, we found a significantly lower prevalence of left main (LM) disease by coronary angiogram (6.1% vs. 33.3%, *p* = 0.007), and also a trend toward higher baseline kynurenine (5.07 vs. 0.79 µM/g, *p* = 0.082) and higher IDO (0.15 vs. 0.02, *p* = 0.081) in patients who survived.

**Conclusion:**

Immunometabolic response mediated via IDO function was enhanced in patients with CAD, and correlated with the extent and severity of disease. Patients with LM disease had higher 1-year mortality. Lower level of IDO, as suggested by inadequate IDO response, demonstrated a trend toward predicting 1-year mortality.

*Trial registration *TCTR Trial registration number TCTR20200626001. Date of registration 26 June 2020. “Retrospectively registered”.

## Introduction

Cardiovascular diseases (CVDs) are the leading cause of mortality and disability worldwide. Acute coronary syndrome (ACS), which is one of the most common presentations of CVD, is triggered by inflammation that causes the rupture of an atherosclerotic lesion, which leads to acute coronary thrombosis and cardiac death. Atherosclerosis is initiated by a maladaptive immune response that is triggered by accumulation and retention of low-density lipoprotein (LDL) in the subendothelial space of large- and medium-sized arteries, which leads to chronic vascular inflammation that is mediated by the activation of immune and vascular cells [1]. The combined effect of activated macrophages and T lymphocytes promotes disease progression. This interaction is present within the atherosclerotic lesion microenvironment with cytokine involvement. The helper T cell (Th1) cytokine interferon γ (IFN-γ) is highly expressed in atherosclerotic lesions, and it mediates immunosuppression in Th1 immune response [2].

Indoleamine 2,3 dioxygenase (IDO) is the first rate-limiting enzyme of the kynurenine (Kyn) pathway of tryptophan (Trp) degradation, and its downstream metabolites can mediate anti-inflammatory and tolerance mechanisms to limit collateral damage and facilitate tissue healing [3]. Extensive research implicates IDO in infection, autoimmune and allergic diseases, cancer, transplantation, and atherosclerosis [4, 5]. In the context of inflammation-driven pathobiological mechanism, there is impairment in IDO activity that exacerbates underlying inflammatory pathology, and reversal of this defect causes improvement [6, 7]. IDO expression is tightly controlled and confined to certain cell types, including endothelial cells, smooth muscle cells, and leukocytes present in the atherosclerotic wall. IDO expression and its catabolic activity are enhanced in response to IFN-γ, which mediates plaque destabilization and acute coronary syndrome [2, 8]. IDO is involved in atherosclerosis as demonstrated by inhibition of IDO boosting innate immune response in the vascular wall and altering lipoprotein metabolism, both of which are known to influence the disease [5]. In addition, IDO mediates anti-inflammatory response in the early phase of atherosclerosis [9]. Recent studies addressed the role of IDO in patients with stable CAD by demonstrating association between IDO activity expressed as kynurenine to tryptophan ratio and adverse outcomes [10, 11]. However, changes in IDO response in patients with ACS, and its association with the extent of coronary involvement and degree of stenosis remained unknown. We hypothesized that a progressive increase in IDO activity correlates with a gradable extent of coronary artery stenosis. The study aims to assess the correlation between IDO activity and the severity of CAD.

## Materials and methods

The Siriraj Institutional Review Board (SIRB) of the Faculty of Medicine Siriraj Hospital, Mahidol University approved this study (COA no. Si 354/2016), the study protocol conforms to the ethical guidelines of the 1975 Declaration of Helsinki, and all included patients gave informed written consent to participate. This prospective cohort study enrolled 305 consecutive patients in whom CAD was suspected, and they all underwent coronary angiogram during January 2017 to June 2019. We excluded patients who might have other non-cardiac causes of elevated inflammatory biomarkers, such as rheumatoid arthritis, allergic asthma, cancer, gingival fibroblasts, neuropathy of Alzheimer’s disease, post-transplantation, inflammatory bowel disease, and active infections. We also excluded patients who refused to participate in the study, which involved consenting to blood testing at baseline and at the 6-month follow-up. Baseline clinical characteristics, presenting symptoms, previous history of cardiovascular intervention, basic laboratory test results, current medication used, and coronary angiogram results were obtained.

### Measurement of high-sensitivity troponin T (hs-TnT) and high-sensitivity C-reactive protein (hs-CRP)

Blood samples for determination of hs-TnT and hs-CRP levels were obtained at baseline before coronary angiography in lithium heparin vacutainer tubes, and then transported to our center’s Central Laboratory (certified by ISO 15189 accreditation). Plasma hs-TnT levels were measured by an immunoassay using electrochemiluminescence technology (Elecsys Troponin T hs STAT; Roche Diagnostics, Rotkreuz, Switzerland) on a cobas 8000 (e 602) analyzer during January 2017 to June 2019 (Elecsys Troponin T hs STAT e602: Limit of blank: 3 pg/mL, Limit of detection: 5 pg/mL, and Limit of quantification: 13 pg/mL), and on a cobas 8000 (e 801) analyzer during December 2017 to June 2019 (Elecsys Troponin T hs STAT e801: Limit of blank: 2.5 pg/mL, Limit of detection: 3 pg/mL, and Limit of quantification: 13 pg/mL). Hs-CRP concentrations were measured by latex-enhanced immunoturbidimetric assays (Roche Diagnostics, Rotkreuz, Switzerland) on a cobas 8000 (c 502) analyzer. The assay has a lower limit of detection of 0.15 mg/L, and an interassay coefficient of variation < 10% at concentrations of 0.3 mg/L. Method validation of the assays was performed according to CLSI EP 15-A3, CLSI EP 09-A3, and CLSI EP 06-A3 guidelines.

### Liquid chromatography–mass spectrometry (LC–MS) analysis of L-tryptophan (L-Trp) and L-kynurenine (L-Kyn)

The kynurenine (Kyn) to tryptophan (Trp) ratio (KTR) was used as a surrogate indicator of IDO activity. Blood samples for determination of Kyn and Trp levels were obtained at baseline before coronary angiogram and at the 6-month follow-up. Indoleamine 2,3 dioxygenase (IDO) activity as reflected by Kyn/Trp ratio was calculated. Kyn and TRP were detected according to the method described by Yong and Lau [12], with minor modifications using high-performance liquid chromatography (HPLC) (Thermoseparation products with UV/VIS Detectors, Fremont, CA, USA). Serum Kyn and Trp levels were measured by reversed-phase HPLC. After overnight fasting, 2 cc of venous was blood drawn from each participant. Collected samples were centrifuged at 2,000 revolutions per minute (rpm) for 10 min within 30 min of collection, and the sample was stored at -80 degrees Celsius until analysis. Frozen serum was thawed at room temperature (RT). The thawed sample was deproteinized by adding an equal volume of 5% (v/v) perchloric acid. The acidified serum was vortexed, allowed to sit at RT for 10 min to precipitate the protein, and was then centrifuged for 10 min at 9000 rpm. Twenty µL of supernatant was injected into the HPLC column for analysis. Tryptophan, kynurenine, and kynurenine acid were determined by reversed-phase HPLC, as previously described [12].

Coronary angiogram was performed according to standard protocol. Two cardiologists who were unaware of the biomarker results reviewed and interpreted the results of coronary angiogram. Significant CAD was defined as visibly obvious luminal coronary obstruction > 70% in at least 1 major epicardial vessel. The percent and degree of coronary artery stenosis was recorded. Location of significant CAD also obtained according to American Heart Association classification. Percutaneous coronary intervention (PCI) or coronary artery bypass graft (CABG) was performed if needed according to standard protocol. The clinical outcome of myocardial infarction, recurrent angina, hospitalization from angina, revascularization, and death at 1 year was prespecified.

### Statistical analysis

Sample size was calculated based on the study from Wirleitner [13]. The mean of IDO activity level in healthy control and patient with significant CAD was 28.1 $\pm$ 5.15 and 36.3 $\pm$ 13.0 µmol/mmol. We assumed the significant CAD patients has mean IDO activity level 20% greater than healthy control (28.1 × 1.2 = 33.72 µmol/mmol). P-values less than 0.05 were considered to indicate significant differences and the power of test was 90%. The proportion in healthy control’s group to significant CAD patient’s group was 1: 3. A total of 304 samples were obtained by 76 of healthy controls and 228 of significant CAD patients. Categorical data were presented as frequency and percentage. Continuous variables were reported as mean ± standard deviation or median (minimum, maximum) depending on the distribution of data. Categorical data were compared using chi-square test or Fisher’s exact test, and continuous data were compared using Student’s *t*-test (normality) or Mann–Whitney U test (non-normality). Patient survival was estimated using Kaplan–Meier survival analysis, and log-rank test was used to compare survival between groups. The best cut-off value for the statistical significance of variables was determined by receiver operating characteristic (ROC) curve analysis, and validity was assessed by the area under the curve (AUC). A *p*-value of less than 0.05 was considered statistically significant. All statistical analyses were performed using PASW Statistics v.22.0 (SPSS, Inc., Chicago, IL, USA).

## Results

### Recruitment and baseline characteristics

A total of 305 patients were enrolled in this study. Of those, 227 had significant CAD, and 78 had non-significant CAD. Ninety-eight patients (32%) presented with recent ACS as the indication for coronary angiogram. Baseline demographic and clinical characteristics of patients are shown in Table 1. Baseline IDO activity, kynurenine level, hs-CRP level, and hs-TnT level were compared between patients with and without CAD.

**Table 1:** Baseline demographic and clinical characteristics

Characteristics	N = 305
Age (years)	63.7 ± 10.0
Male sex	187 (61.3%)
Creatinine (mg/dL)	1.07 (0.34, 10.15)
hs-TnT (ng/l)	13.20 (3.09, 294.20)
hs-CRP (mg/l)	1.47 (0.15, 130.05)
Kynurenine (µM)	4.94 (0.00, 11,731.00)
Tryptophan (µM)	36.99 (0.00, 43,111.29)
IDO activity	0.14 (0.00, 4.58)
Diabetes	134 (43.9%)
Hypertension	272 (89.2%)
Dyslipidemia	233 (76.4%)
*Recent ACS*	
STEMI	31 (10.2%)
Non STEMI	53 (17.4%)
Unstable angina	14 (4.6%)
Stable CAD	114 (37.4%)
Other (Pre-op valvular HD / HF / Reduced EF)	93 (30.5%)
Ischemic stroke	13 (4.3%)
Peripheral vascular disease	14 (4.6%)
*Current medications*	
ASA	269 (88.2%)
Plavix	183 (60.0%)
Beta blockers	245 (80.3%)
Statin	258 (84.6%)
*Baseline coronary angiography*	
Nonsignificant CAD	78 (25.6%)
Single-vessel disease	81 (26.6%)
Double-vessel disease	66 (21.6%)
Triple-vessel disease	80 (26.2%)
*Treatment following baseline coronary angiography*	
Medication only	123 (40.3%)
PCI	145 (47.5%)
CABG	37 (12.1%)

Data presented as frequency and percentage, mean ± standard deviation, or median (minimum, maximum)

hs-TnT, high-sensitivity troponin T; CRP, C-reactive protein; IDO, indoleamine 2,3 dioxygenase; ACS, acute coronary syndrome; STEMI, ST-segment elevation myocardial infarction; CAD, coronary artery disease; HD, heart disease; HF, heart failure; EF, ejection fraction; ASA, Aspirin; CAD, coronary artery disease; PCI, percutaneous coronary intervention; CABG, coronary artery bypass graft

**Fig. 1:**
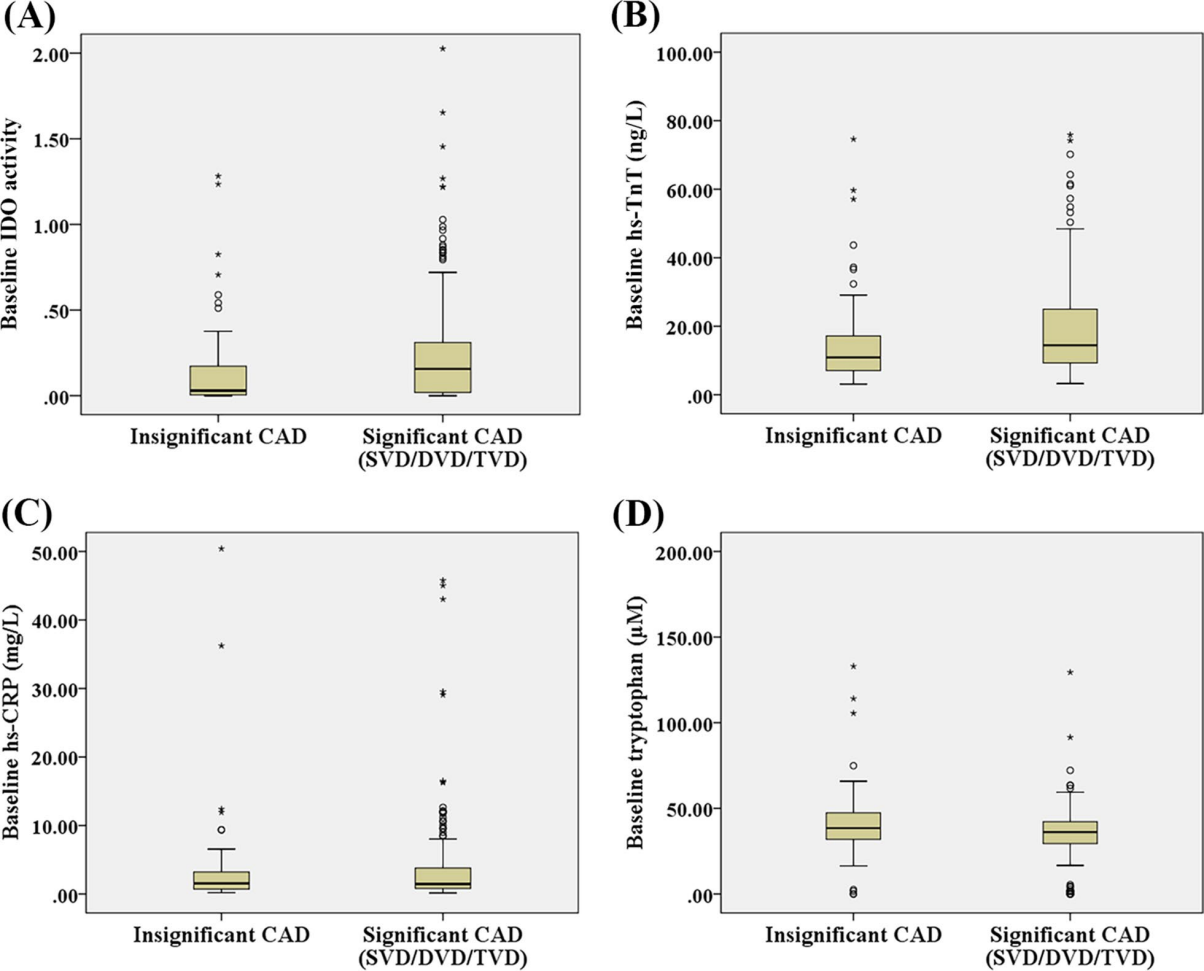
**A** Box plot of indoleamine 2,3 dioxygenase (IDO) activity values at baseline in the insignificant coronary artery disease (CAD) group, and in the significant CAD [single-vessel disease (SVD)/double-vessel disease (DVD)/triple-vessel disease (TVD)] group. **B** Box plot of high-sensitivity troponin T (hs-TnT) values at baseline in the insignificant CAD group, and in the significant CAD (SVD/DVD/TVD) group. **C** Box plot of high-sensitivity C-reactive protein (hs-CRP) values at baseline in the insignificant CAD group, and in the significant CAD (SVD/DVD/TVD) group. **D** Box plot of tryptophan values at baseline in the insignificant CAD group, and in the significant CAD (SVD/DVD/TVD) group

The levels of baseline IDO activity and kynurenine-levels in patients with significant CAD presenting with single-, double-, or triple-vessel involvement were significantly higher than in individuals without significant CAD (0.16, 0.13, and 0.17 vs. 0.03, respectively; *p* = 0.003; and, 5.24, 4.58, and 5.89 vs. 2.74 µM/g, respectively; *p* = 0.011) (Fig. 1A), and a similar significant increase in baseline hs-TnT levels was also observed (12.86, 12.22, and 18.27 vs. 10.89 mg/dL, respectively; *p* < 0.001) (Fig. 1B). In addition, the degree of IDO activity was directly associated with the severity of coronary stenosis (rs = 0.154, *p* = 0.007). Significant correlation was found between degree of IDO activity and the degree of RCA stenosis, but not for other anatomical distribution of coronary arterial stenosis (rs = 0.169, *p* = 0.003 for RCA; rs = 0.062, *p* = 0.284, for LAD; and, rs = 0.093, *p* = 0.106 for LCX). However, we failed to demonstrate significant changes in hs-CRP and tryptophan levels in subjects with significant CAD compared to the insignificant CAD group [(1.31, 1.51, and 1.47 vs. 1.55 mg/dL, respectively; *p* = 0.742) (Fig. 1C), and (37.94, 34.31, and 35.63 vs. 38.43 µM/g, respectively; *p* = 0.096) (Fig. 1D)].

### Cutoff point of baseline IDO activity, kynurenine, and hs-TnT levels for predicting significant coronary artery disease (CAD)

#### IDO activity

**Fig. 2:**
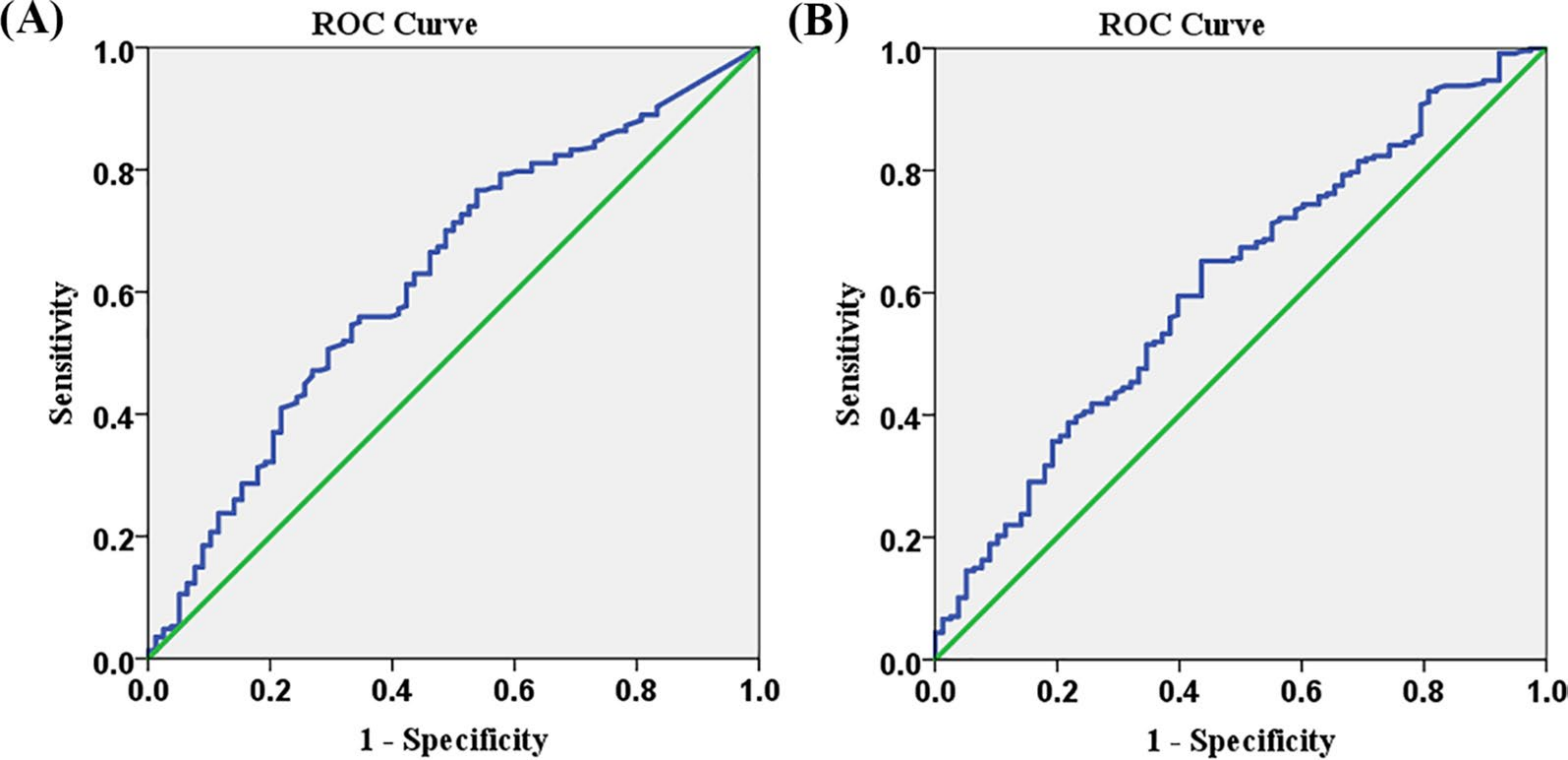
**A** Receiver operating characteristic (ROC) curve of baseline indoleamine 2,3 dioxygenase (IDO) activity for significant coronary artery disease (CAD) prediction. **B** ROC curve of baseline high-sensitivity troponin T (hs-TnT) for significant CAD prediction

Receiver operating characteristic (ROC) curve analysis showed that baseline IDO activity levels ≥ 0.0174 had a sensitivity of 76.7% (range: 70.7–81.7%) and specificity of 46.2% (range: 35.5–57.1%) for predicting the presence of significant CAD. The ROC curve yielded an area under the curve (AUC) of 0.626 (*p* = 0.001) (Fig. 2A).

#### hs-TnT levels

The ROC curve showed that baseline hs-TnT levels ≥ 11.39 pg/mL had a sensitivity of 65.2% (range: 58.6–71.4%) and specificity of 56.4% (range: 44.7–67.6%) for predicting significant CAD. The ROC curve yielded an AUC of 0.614 (*p* = 0.003) (Fig. 2B).

### Clinical endpoints based on baseline demographic and biomarkers

**Fig. 3:**
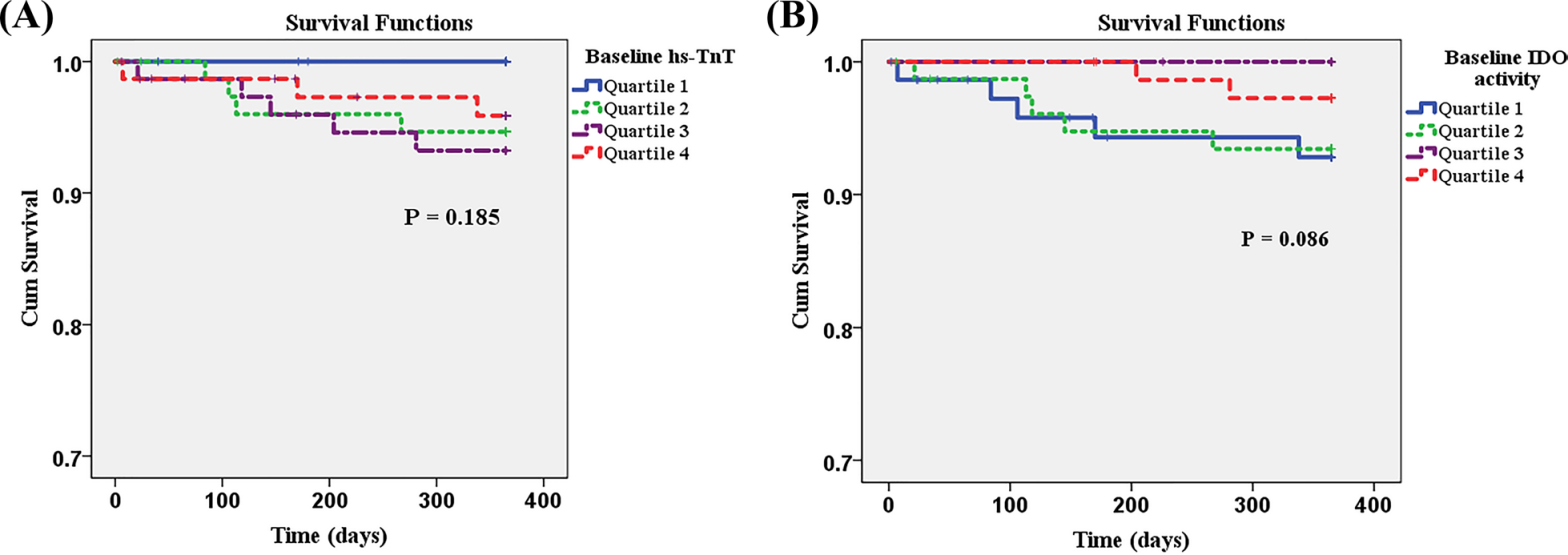
**A** Survival at 1 year according to quartiles of baseline high-sensitivity troponin T (hs-TnT) levels. **B** Survival at 1 year according to quartiles of indoleamine 2,3 dioxygenase (IDO) activity levels

Twelve patients expired within 1 year of the longitudinal follow-up. Six patients had CV-related death, including 2 myocardial infarction, 3 heart failure, and 1 aortic dissection. The other six patients had non-CV-related death, including 3 sepsis, 2 malignant neoplasm, and 1 gastrointestinal bleeding. The patients who died had a higher incidence of LM disease than survivors (33.3% vs. 6.1%, *p* = 0.007) (Table 2). There was a trend toward higher kynurenine (5.07 vs. 0.79 µM/g, *p* = 0.082) and higher IDO activity (0.15 vs. 0.02, *p* = 0.081) levels in the survivor group compared to those who died. Patients who experienced combined cardiac adverse events (death and/or myocardial infarction and/or recurrent chest pain) had lower kynurenine (1.97 vs. 5.23 µM/g, *p* = 0.036) and higher hs-TnT (16.92 vs 12.88 pg/mL, p = 0.038) levels than those who had no event (Table 3). Patients with less severe atherosclerosis (insignificant CAD and single-vessel disease) who experienced combined cardiac adverse events exhibited lower baseline kynurenine levels and IDO activity than those who had no events (0.38 vs. 4.94, *p* = 0.017; and, 0.01 vs. 0.14, *p* = 0.023, respectively). This was, however, not the case for patients with more severe atherosclerosis (double- and triple-vessel disease) (Table 3). Figure 3A, B show survival according to quartiles of baseline hs-TnT and IDO activity level.

**Table 2:** Comparison of post-catherization diagnosis and biomarkers between patients who had survived and patients who had died by the 1-year follow-up

Parameters	Survived (n = 293)	Died (n = 12)	*p*-value
*All patients (N* = *305)*			
Baseline hs-TnT	12.96 (3.09, 294.20)	15.89 (10.41, 61.03)	0.154
Baseline Kynurenine	5.07 (0.00, 11,731.00)	0.79 (0.00, 32.84)	0.082
Baseline IDO activity	0.15 (0.00, 4.58)	0.02 (0.00, 0.79)	0.081

Insignificant CAD/SVD patients (n = 159)	Survived (n = 155)	Died (n = 4)	
Baseline hs-TnT	11.53 (3.09, 141.50)	13.24 (12.89, 16.92)	0.492
Baseline Kynurenine	4.68 (0.00, 7892.33)	0.38 (0.00, 0.82)	***0.037***
Baseline IDO activity	0.13 (0.00, 4.13)	0.01 (0.00, 0.02)	***0.040***

DVD/TVD patients (n = 146)	Survived (n = 138)	Died (n = 8)	
Baseline hs-TnT	15.93 (4.53, 294.20)	17.63 (10.41, 61.03)	0.355
Baseline Kynurenine	5.58 (0.00, 11,731.00)	2.28 (0.39, 32.84)	0.380
Baseline IDO activity	0.16 (0.00, 4.58)	0.06 (0.01, 0.79)	0.380

Data presented as frequency and percentage or median (minimum, maximum)

A *p*-value < 0.05 indicates statistical significance

CAD, coronary artery disease; SVD, single-vessel disease; LM, left main; hs-TnT, high-sensitivity troponin T; IDO, indoleamine 2,3 dioxygenase; DVD, double-vessel disease; TVD, triple-vessel disease

**Table 3:** Comparison of post-catherization diagnosis and biomarkers between patients who had no event and patients who had combined events by the 1-year follow-up

Parameters	No event (n = 274)	Combined events (n = 17)	*p*-value
*All patients (N* = *291)*			
Baseline hs-TnT	12.88 (3.09, 294.20)	16.92 (8.28, 124.90)	***0.038***
Baseline Kynurenine	5.23 (0.00, 11,731.00)	1.97 (0.00, 32.84)	***0.036***
Baseline IDO activity	0.15 (0.00, 4.58)	0.05 (0.00, 2.91)	0.159

Insignificant CAD/SVD patients (n = 152)	No event (n = 146)	Combined events (n = 6)	
Baseline hs-TnT	11.59 (3.09, 141.50)	15.13 (12.89, 124.90)	0.111
Baseline Kynurenine	4.94 (0.00, 7892.33)	0.38 (0.00, 4.45)	***0.017***
Baseline IDO activity	0.14 (0.00, 4.13)	0.01 (0.00, 0.14)	***0.023***

DVD/TVD patients (n = 139)	No event (n = 128)	Combined events (n = 11)	
Baseline hs-TnT	15.05 (4.53, 294.20)	17.13 (8.28, 61.03)	0.290
Baseline Kynurenine	5.70 (0.00, 11,731.00)	4.37 (0.38, 32.84)	0.264
Baseline IDO activity	0.16 (0.00, 4.58)	0.12 (0.01, 2.91)	0.767

Data presented as frequency and percentage or median (minimum, maximum)

A *p*-value < 0.05 indicates statistical significance

Combined events: All cause death or myocardial infarction or recurrent chest pain

CAD, coronary artery disease; SVD, single-vessel disease; LM, left main; hs-TnT, high-sensitivity troponin T; IDO, indoleamine 2,3 dioxygenase; DVD, double-vessel disease; TVD, triple-vessel disease

### Limitations of IDO in detected significant CAD

We found that baseline level of kynurenine and IDO activity also elevated in the presence of heart failure and diabetes mellitus without significant CAD. The levels of baseline IDO activity and kynurenine-levels in patients with significant CAD were significantly higher than in individuals without significant CAD even we have excluded patients with heart failure or diabetes mellitus. The correlation of kynurenine and IDO activity levels and severity of CAD in the presence of heart failure, diabetes mellitus and valvular heart disease were presented in Table 4.

**Table 4:** The correlation of kynurenine and IDO activity levels and severity of CAD in the presence of heart failure, diabetes mellitus and valvular heart disease

	Insignificant CAD (n = 78)	Significant CAD (n = 227)	*p*-value
*All patients (n* = *305)*			
Baseline Kynurenine	2.74 (0.00, 70.58)	5.36 (0.00, 11,731.00)	***0.007***
Baseline IDO activity	0.03 (0.00, 3.78)	0.16 (0.00, 4.58)	***0.001***

Excluded pre-op for VHD (n = 279)	n = 59	n = 220	
Baseline Kynurenine	1.00 (0.00, 70.58)	5.37 (0.00, 11,731.00)	***0.005***
Baseline IDO activity	0.02 (0.00, 3.78)	0.16 (0.00, 4.58)	***0.001***

In presence of HF (n = 66)	n = 41	n = 25	
Baseline Kynurenine	4.97 (0.00, 70.58)	1.21 (0.00, 44.63)	0.358
Baseline IDO activity	0.09 (0.00, 3.78)	0.03 (0.00, 1.22)	0.512

Excluded HF (n = 239)	n = 37	n = 202	
Baseline Kynurenine	0.82 (0.00, 42.74)	5.62 (0.00, 11,731.00)	***0.002***
Baseline IDO activity	0.01 (0.00, 1.28)	0.17 (0.00, 4.58)	** < *0.001***

In presence of DM (N = 134)	n = 25	n = 109	
Baseline Kynurenine	4.60 (0.00, 34.41)	4.90(0.00, 11,731.00)	0.276
Baseline IDO activity	0.11 (0.00, 0.71)	0.15 (0.00, 3.71)	0.107

Excluded DM (n = 171)	n = 53	n = 118	
Baseline Kynurenine	1.84 (0.00, 70.58)	5.73(0.00, 10,639.00)	***0.009***
Baseline IDO activity	0.02 (0.00, 3.78)	0.16 (0.00, 4.58)	***0.002***

Data are presented as median(minimum,maximum)

A *p*-value < 0.05 indicates statistical significance

CAD, Coronary artery disease; VHD, Valvular heart disease; HF, Heart failure; DM, Diabetes Mellitus; IDO, Indoleamine 2,3 dioxygenase

## Discussion

In this study, we showed that gradable immunometabolic response of IDO to the proinflammatory activity of atherosclerosis was enhanced by more advanced CAD. Patients with significant single-, double-, and triple-vessel disease in combination had markedly increased IDO activity and kynurenine levels compared to individuals with insignificant CAD. In addition, hs-TnT levels in the significant stenosis group were greater than in the latter control group, and this mirrored active and advanced atherosclerosis in subjects with chronic stable CAD, which accounts for the vast majority of subjects in this study. The magnitude of incremental IDO activity response significantly correlated with the degree of coronary stenosis, particularly at the right coronary artery, which suggests the intensity of counter-regulatory immunotolerance mechanisms elicited in an attempt to maintain immunohomeostasis for ongoing low-grade inflammation in the vascular wall of chronic advanced stable CAD. However, the maintenance of IDO activity had a tendency to decline in CAD patients who underwent multiple coronary adverse events (cardiac death, myocardial infarction, and recurrent cardiac chest pain) compared with those suffering a single event (*p* > 0.05), which indicates impairment of IDO response to some extent.

Proinflammatory cytokine-driven active inflammation in atherosclerosis elicits a strong IDO response [14]. Interferon-gamma (IFN-γ) exemplifies the induction of IDO activity in atherosclerotic vascular wall [15]. This is also the case for other proinflammatory cytokines involved in inflammatory signaling to vascular dysfunction in atherosclerosis, including tumor necrosis factor (TNF) and interleukin-1 (IL-1) [4]. An increasing body of evidence indicates that IDO promotes immune tolerance, decreases inflammation, and functions as a homeostatic mechanism against excessive inflammatory reactions. Therefore, an intact counter-regulatory IDO response may explain our observation that hs-CRP levels failed to significantly elevate in patients with significant CAD, whereas IDO activity and kynurenine levels both increased. In addition, increased IDO response with higher kynurenine concentrations was found to associate with an increase in myocardial ischemic events in significant CAD individuals, which was reflected by the significant increase in hs-TnT levels. This leads us to hypothesize that positive IDO response may benefit ischemic myocardium since IDO can increase the proportion of regulatory T cells (Tregs) in relation to enhancement of T cell apoptosis [16]. Despite the requirement for translational studies to further test this hypothesis, Tregs directly protected cardiomyocytes against apoptosis via cell–cell contact, enriched IL-10 microenvironment, and inhibited proinflammatory cytokines released from ischemic cardiomyocytes [17]. Moreover, the protective effects of Tregs on vulnerable myocardium were extended to myocardial ischemia/reperfusion injury [18, 19].

Previous studies demonstrated that IDO activity was associated with atherosclerotic risk factors encompassing hypertension, diabetes, and dyslipidemia [9, 20, 21], all of which including hs-TnT predicted the infliction of significant coronary disease as demonstrated by coronary angiogram. However, it remains unknown whether the predictive power of atherosclerotic risk factors works independently or is dependent upon IDO function in a temporal manner. It is also not exactly known whether the composite or separate use of atherosclerotic risk factors with these biomarkers would provide the most benefit for prediction. The present study indicated the possibility that the sustainability of IDO response seemed to be affected by multiple hits on afflicted atherosclerotic coronary vessels, with decline in kynurenine levels in response to multiple cardiac adverse events. Therefore, further investigation into the mechanisms underlying the decrease in long-term IDO response with associated clinical characteristics is clearly warranted.

Previous reports showed that elevated plasma and urine IDO predicted major adverse cardiovascular events (MACE) and mortality in stable CAD patients [10, 11]. However—in our study, a lower level of IDO activity was more predictive of a 1-year MACE or mortality than a higher level of IDO activity. The plausible explanation for this difference between our study and previous studies may be the fact that we recruited a study population that included a mixture of both stable CAD and ACS patients. We speculated that the observed alternating IDO response to suppress acute atherosclerotic vascular inflammation in recurrent ACS may compromise its ability to resume IDO production, which resulted in our observed absence of association between inadequately rising IDO activity levels and a reduced incidence of a MACE. Our original aim was to measure IDO activity levels at baseline and at the 6-month follow-up; however, many of our study participants died during the 6-month follow-up period. This explains our failure to obtain sufficient plasma samples at 6-month time point for IDO activity measurements. Therefore, further investigation into the patterns of IDO activity increments in a large number of patients is clearly warranted to test our hypothesis.

Several limitations should be aware for practical use of IDO activity to detect significant CAD. Even though statistically significant, the discriminatory accuracy of baseline IDO activity in classifying significant CAD seemed suboptimal (AUC 0.626). IDO activity level ≥ 0.0174 has a good sensitivity but low specificity for predicting the presence of significant CAD. In the setting that non-cardiac causes of elevated inflammatory biomarkers were excluded, diabetes mellitus and heart failure which are associated with chronic inflammation could have an elevated IDO activity level without the presence of significant CAD.

## Study limitations

This prospective cohort study recruited both stable CAD and recent ACS patients who were scheduled to undergo coronary angiogram in the cardiac catheterization laboratory. The sample size was calculated based on the power to detect significant CAD, but not a MACE. Blood sampling at baseline and at 6 months was also a criterion that patients had to agree to in order to be included. Patients who refused to participate in the research protocol were excluded. We also excluded non-cardiac cause that known to elevated inflammatory biomarkers. Our study protocol was written in 2016 before the information of elevated IDO activity in heart failure setting [22, 23] and metabolic syndrome setting [24]. We included 66 patients with heart failure and 134 patients with diabetes mellitus to our study. We still found a significant different of IDO activity level in the presence of significant CAD even we excluded patients with heart failure patients or diabetes mellitus (Table 4). We foresee how the rise and fall pattern of IDO activity at baseline and 6 months could also help us to understand how IDO activity plays a role in risk prediction. The rise and fall pattern of IDO activity at baseline and at 6 months helps us to better understand how IDO activity plays a role in risk prediction because IDO activity is both modulated by inflammation and functions to suppress inflammation. However, most death and CV-related death occurred before the 6-month follow-up. Therefore, the number of patients was too small to draw any conclusions about different patterns of change in hs-TnT, kynurenine, and IDO activity between patients with stable CAD and recent ACS.

## Conclusions

Immunometabolic response mediated via IDO function was enhanced in patients with CAD, and correlated with the severity and extent of disease. IDO activity has a good sensitivity but low specificity for detecting significant CAD. Lower level of IDO activity, as suggested by inadequate IDO response, demonstrated a trend toward predicting 1-year mortality. Our findings provide a more comprehensive understanding of IDO activity, kynurenine, and hs-TnT in clinical practice.

## Data Availability

The datasets used and/or analyzed during the current study are available from the corresponding author on reasonable request. Identifying/confidential patient data should not be shared.

## Abbreviations

IDO: Indoleamine 2,3 dioxygenase
Kyn: Kynurenine
Trp: Tryptophan
KTR: Kynurenine to tryptophan ratio
CAD: Coronary artery disease
hs-CRP: High-sensitivity C-reactive protein
hs-TnT: High-sensitivity troponin T
ACS: Acute coronary syndrome
LM: Left main
RCA: Right coronary artery
LAD: Left anterior descending artery
LCX: Left circumflex artery
CVDs: Cardiovascular diseases
LDL: Low-density lipoprotein
Th1: Helper T cell
IFN-γ: Interferon γ
HPLC: High-performance liquid chromatography
PCI: Percutaneous coronary intervention
CABG: Coronary artery bypass graft
MACE: Major adverse cardiovascular events
SVD: Single-vessel disease
DVD: Double-vessel disease
TVD: Triple-vessel disease
